# Population Pharmacokinetic Modeling of Bedaquiline among Multidrug-Resistant Pulmonary Tuberculosis Patients from China

**DOI:** 10.1128/aac.00811-22

**Published:** 2022-09-15

**Authors:** Jin Zou, Shuyan Chen, Weiqiao Rao, Liang Fu, Jiancong Zhang, Yunli Liao, Ying Zhang, Ning Lv, Guofang Deng, Shijin Yang, Liang Lin, Lujin Li, Siqi Liu, Jiuxin Qu

**Affiliations:** a Department of Clinical Laboratory, Shenzhen Third People’s Hospital, Southern University of Science and Technology, National Clinical Research Center for Infectious Diseases, Shenzhen, China; b College of Life Sciences, University of Chinese Academy of Sciences, Beijing, China; c Shenzhen Third People’s Hospital, Second Hospital Affiliated to Southern University of Science and Technology, National Clinical Research Center for Infectious Diseases, Shenzhen, China; d BGI-Shenzhen, Shenzhen, China; e Division Two of Pulmonary Diseases Department, Shenzhen Third People’s Hospital, Southern University of Science and Technology, National Clinical Research Center for Infectious Diseases, Shenzhen, China; f Center for Drug Clinical Research, Shanghai University of Traditional Chinese Medicine, Shanghai, China

**Keywords:** population pharmacokinetics, bedaquiline, multidrug-resistant tuberculosis, GGT, rs319952

## Abstract

Bedaquiline has been widely used as a part of combination dosage regimens for the treatment of multidrug-resistant tuberculosis (MDR-TB) patients with limited options. Although the effectiveness and safety of bedaquiline have been demonstrated in clinical trials, limited studies have investigated the significant pharmacokinetics and the impact of genotype on bedaquiline disposition. Here, we developed a population pharmacokinetic model of bedaquiline to describe the concentration-time data from Chinese adult patients diagnosed with MDR-TB. A total of 246 observations were collected from 99 subjects receiving the standard recommended dosage. Bedaquiline disposition was well described by a one-compartment model with first-order absorption. Covariate modeling identified that gamma-glutamyl transferase (GGT) and the single-nucleotide polymorphism (SNP) rs319952 in the *AGBL4* gene were significantly associated with the apparent clearance of bedaquiline. The clearance (CL/*F*) was found to be 1.4 L/h lower for subjects with allele GG in SNP rs319952 than for subjects with alleles AG and AA and to decrease by 30% with a doubling in GGT. The model-based simulations were designed to assess the impact of GGT/SNP rs319952 on bedaquiline exposure and showed that patients with genotype GG in SNP rs319952 and GGT ranging from 10 to 50 U/L achieved the targeted maximum serum concentration at steady state (*C*_max,ss_). However, when GGT was increased to 100 U/L, *C*_max,ss_ was 1.68-fold higher than the highest concentration pursued. The model developed provides the consideration of genetic polymorphism and hepatic function for bedaquiline dosage in MDR-TB adult patients.

## INTRODUCTION

Tuberculosis (TB) has been the leading cause of death due to a single infectious agent worldwide since 2007, although it became the second leading cause of death in 2020 because of COVID-19 (1). China has been listed by WHO as one of the 10 countries in all three lists for “high-burden country” (HBC), for TB, HIV-associated TB, and multidrug-resistant TB (MDR-TB), in the period from 2021 to 2025. MDR-TB is defined as TB that resistant to at least the two most effective first-line drugs, isoniazid and rifampicin, and remains the greatest public health concern (2). The burden of MDR-TB is stable globally, and the rates of MDR-TB were estimated to be 3 to 4% in patients first diagnosed with TB and 18 to 21% of those previously treated for TB (1).

In 2012, bedaquiline (BDQ; formerly TMC207), the first in the new diarylquinoline class of antituberculosis drugs, became the first new drug to be approved by the U.S. Food and Drug Administration (FDA) for MDR-TB treatment in the past 40 years (3). The protonated active form binds to mycobacterial ATP synthase to kill the bacteria by interfering with proton transfer and inducing conformational changes of the ATP synthase (4, 5). In 2014, one phase 2b clinical trial reported that the addition of BDQ to the background regimen resulted in faster culture conversion and more significant culture conversions at 120 weeks compared to the results for the placebo group (6). At present, BDQ is classified in the group A drugs by WHO and recommended for the longer treatment regimens of MDR-TB, which have been used in 109 countries since 2019 (1, 7). The approved regimen of BDQ in adult patients includes 400 mg daily for 2 weeks, followed by 200 mg three times per week for another 22 weeks (8). The pharmacokinetic (PK) profile of BDQ shows that the maximum serum concentration (*C*_max_) is achieved about 5 h after administration, and the effective half-life is approximately 24 h after 2 weeks of 400-mg-daily dosing (8).

BDQ is mainly metabolized into a less active *N*-mono desmethyl metabolite (M2) and an *N*-didesmethyl metabolite (M3) by cytochrome P450 (CYP) isoenzyme CYP3A4, based on clinical trials (8). Other *in vitro* studies also reported that two novel metabolites (M5 and M6) were identified in human hepatocytes and that other CYP isoforms, CYP1A1, CYP2C8, CYP2C18, and CYP2C19, play a role in BDQ metabolism (9, 10). Data from two clinical trials indicated that the use of BDQ could be associated with an increased risk of death, QT interval prolongation, and hepatotoxicity (11). Although the underlying mechanisms of antituberculosis drug-induced hepatotoxicity (ATDH) have not been fully understood, genetic variants have been reported in association with ATDH susceptibility in Chinese and other populations, such as polymorphisms in drug-metabolizing enzymes *N*-acetyltransferase 2 (NAT2) and cytochrome P450 2E1 (CYP2E1) (12–15). In addition, other genetic predictors were suggested to play a role in the risk of ATDH in Chinese TB patients (16). Therefore, we included the analysis of genetic polymorphisms to explore the relationship between BDQ exposure and gene variants.

Three population pharmacokinetics models of BDQ in healthy adults and patients with tuberculosis were recently published (17–19). The first model was based on two clinical trials, including healthy volunteers, patients with drug-sensitive TB, and patients with MDR-TB, which characterized the effects of race and sex on apparent clearance (CL/*F*) and apparent central volume of distribution (*V_c_*/*F*) individually (17). The second model described the PK of BDQ and its metabolite M2 in MDR-TB patients receiving 24 weeks of BDQ treatment. Significant covariates, such as weight, albumin, age, and race, were also characterized (18). A recently published model was developed to describe BDQ PK in Chinese patients with MDR-TB, but the significant covariates were not well characterized (19). In this study, we aimed to develop a population PK model of BDQ in Chinese MDR-TB patients and perform analysis of covariates and the association of genotype polymorphisms with variability in BDQ plasma PK.

## RESULTS

### Study population.

A total of 246 BDQ concentrations were obtained from 99 patients who were recruited from October 2020 to October 2021. All patients were diagnosed with multidrug-resistant tuberculosis and orally administered the approved regimen. One patient provided eight samples, 1 patient provided seven samples, 5 patients provided six samples, 7 patients provided five samples, 11 patients provided four samples, 12 patients provided three samples, 24 patients provided two samples, and 38 patients provided one sample. Blood samples were randomly collected within a dosing interval at the steady-state on therapy that ranged from 2 to 64 weeks before PK sampling (median, 20 weeks). Only one observation was below the limit of detection (LOD) and replaced by the value of LOD in the assay, 0.024 μg/mL. The baseline demographic and clinical characteristics of the study population are summarized in Table 1. The distribution of the single-nucleotide polymorphisms (SNPs) of the 99 patients is shown in Table 2.

**TABLE 1:** Summary of baseline characteristics

Characteristic^*a*^	Mean value (SD)	Range (minimum–maximum)
Age (yr)	38.1 (14.1)	11–78
Height (cm)	166.4 (12.4)	73.3–183
Wt (kg)	58 (12.6)	17–90.7
RBC (10^12^/L)	4.5 (0.7)	2.54–6.32
HGB (g/L)	134.2 (22.6)	67–174
WBC (10^9^/L)	5.9 (2.1)	2.47–13.88
PLT (10^9^/L)	227.1 (79.4)	105–553
NEUT (%)	59.1 (11)	32–91.7
EO (%)	2.6 (1.9)	0–10.1
BASO (%)	0.5 (0.2)	0–1
Lymph (%)	28.2 (9.9)	3.7–52.5
MONO (%)	9.6 (2.4)	4.1–16.8
ALT (U/L)	28.6 (24.2)	6–138.8
AST (U/L)	38.5 (24)	14.5–176.1
GGT (U/L)	36.4 (31.1)	8–271.8
TP (g/L)	77.5 (5.8)	65.4–94.6
ALB (g/L)	46.2 (3.3)	38.2–55.6

a RBC, red blood cell; HGB, hemoglobin; WBC, white blood cell; PLT, platelets; NEUT, neutrophils; EO, eosinophils; BASO, basophils; MONO, monocytes; ALT, alanine transaminase; AST, aspartate transaminase; GGT, gamma-glutamyl transferase; TP, total protein; ALB, albumin.

**TABLE 2:** SNPs in the analysis of 99 patients

SNP	Gene	Allele^*a*^	No. of patients	Allelic frequency (%)
rs1045642	ABCB1	A	11	11.11
		G (Ref)	27	27.27
		GA	45	45.45
		NA	16	16.16
rs3740065	ABCC2	A (Ref)	33	33.33
		AG	40	40.40
		G	10	10.10
		NA	16	16.16
rs319952	AGBL4	A (Ref)	31	31.31
		AG	39	39.39
		G	13	13.13
		NA	16	16.16
rs320003	AGBL4	A	12	12.12
		G (Ref)	69	69.69
		NA	18	18.18
rs2070401	BACH1	A (Ref)	50	50.50
		G	4	4.04
		GA	29	29.29
		NA	16	16.16
rs9332096	CYP2C9	C (Ref)	77	77.78
		CT	6	6.06
		NA	16	16.16
rs4986893	CYP2C19	AG	7	7.07
		G (Ref)	76	76.77
		NA	16	16.16
rs2031920	CYP2E1	C (Ref)	46	46.46
		T	3	3.03
		TC	12	12.12
		NA	38	38.38
rs1695	GSTP1	A (Ref)	53	53.54
		G	1	1.01
		GA	28	28.28
		NA	17	17.17
rs11080344	NOS2	C	29	29.29
		T (Ref)	18	18.18
		TC	36	36.36
		NA	16	16.16
rs10946739	RIPOR2	C (Ref)	55	55.56
		TC	28	28.28
		NA	16	16.16
rs4149056	SLCO1B1	C	8	8.08
		T (Ref)	59	59.60
		TC	15	15.15
		NA	17	17.17
rs1495741	Unknown	A	18	18.18
		AG	38	38.38
		G (Ref)	26	26.26
		NA	17	17.17
rs11125883	XPO1	A (Ref)	30	30.30
		C	13	13.13
		CA	40	40.40
		NA	16	16.16

a NA, not applicable.

### Model building.

The population pharmacokinetics of BDQ was best characterized by a one-compartment model. The model was parameterized in terms of the apparent central volume of distribution (*V_c_*/*F*), apparent clearance (CL/*F*), and absorption rate constant (*K_a_*). The residual variability was best described by the proportional model.

**FIG 1:**
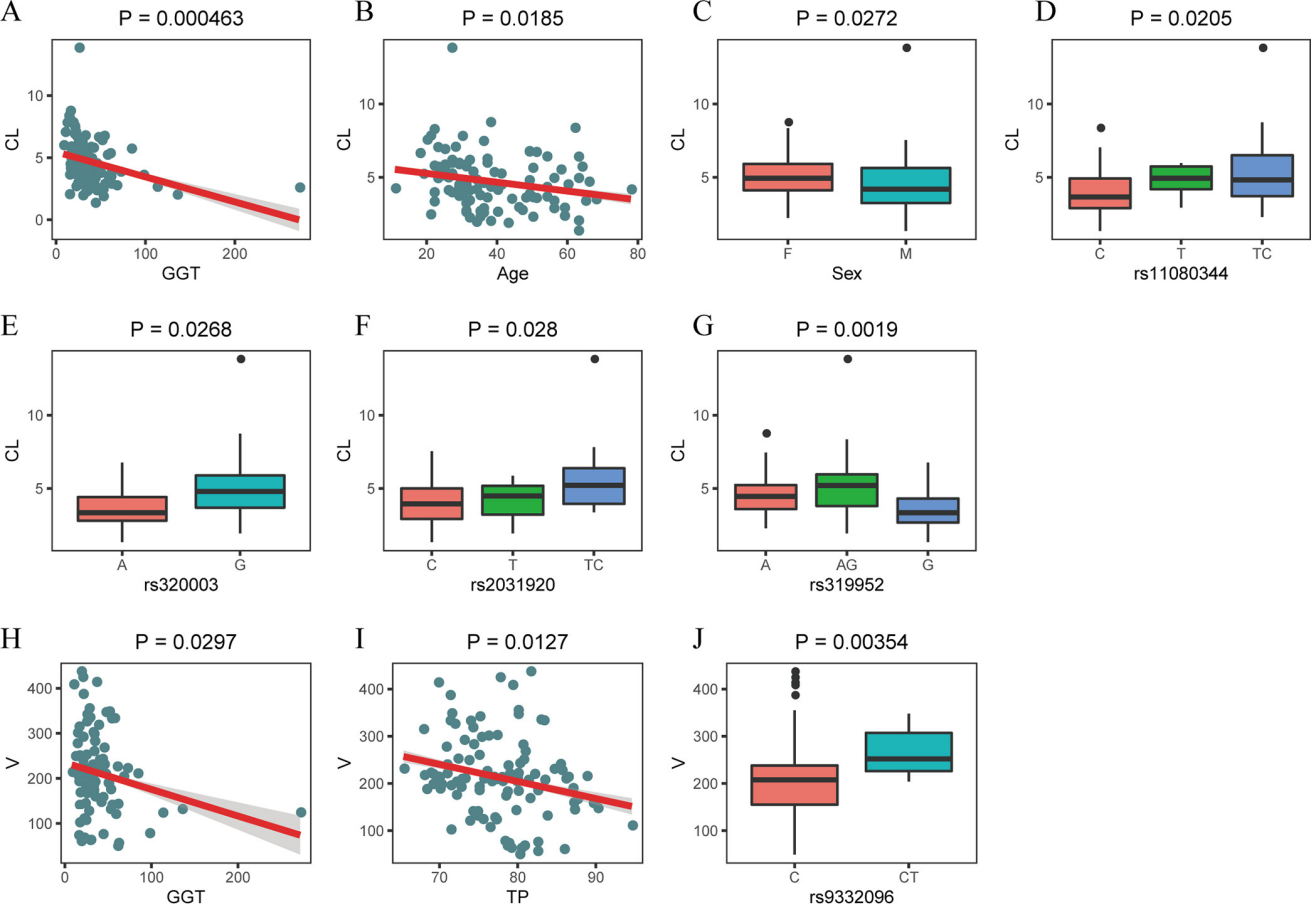
Graphical analysis of covariates that were significantly related to CL/*F* and *V_c_*/*F*.

Based on graphical analysis, 7 covariates were found to be significantly related to CL/*F*, including age (*P* = 0.0185), sex (*P* = 0.0272), gamma-glutamyl transferase (GGT) concentration (*P* = 0.000463), and SNPs rs320003 (AA versus GG) (*P* = 0.0268), rs2031920 (CC versus TC&TT) (*P* = 0.028), rs11080344 (CC versus TC&TT) (*P* = 0.0205), and rs319952 (GG versus AA&AG) (*P* = 0.0019), and 3 covariates were significantly related to *V_c_*/*F*, including GGT (*P* = 0.0297), total protein (TP) (*P* = 0.0127), and SNP rs9332096 (CC versus CT) (*P* = 0.00354) (Fig. 1). After a forward inclusion step, we found that GGT and SNP rs319952 significantly affected CL/*F* (*P* < 0.01) and TP had significant effects on *V_c_*/*F* (*P* < 0.01). In the backward elimination step, only GGT and rs319952 were retained in the final model (*P* < 0.005). The final covariate model was as follows: for rs319952 genotype A&AG,

(1) $$\text{CL/}F=4.54\times {\left(\text{GGT/28}.9\right)}^{-0.476}$$

and for rs319952 genotype G,

(2) $$\text{CL/}F=4.54\times {\left(\text{GGT/28}.9\right)}^{-0.476}\;-\;1.4$$

The results showed that the higher the GGT, the lower the CL/*F*, and when the GGT increased by 30 U/L, the CL/*F* decreased by 30%; when the genotype of rs319952 was G, CL/*F* decreased by 1.4 L/h compared with A&AG. The parameter estimates of the final model are shown in Table 3.

**TABLE 3:** Parameter estimates for the final model

Parameter^*a*^	Parameter estimate RSE (%)^*b*^	Bootstrap analysis median value (5th–95th percentile)^*c*^	% shrinkage
Pharmacokinetic parameter			
*K_a_* (h^−1^)	0.447 (16.6)	0.443 (0.348 to 0.598)	
CL/*F* (L/h)	4.54 (5.3)	4.52 (4.13 to 4.93)	
*V_c_*/*F* (L)	227 (16.8)	226 (179 to 316)	
Covariate parameter			
θGGT on CL/*F*	−0.476 (18.1)	−0.486 (−0.645 to −0.340)	
θrs319952 on CL/*F*	−1.4 (27.1)	−1.35 (−1.994 to −0.662)	
Interindividual variability (%)			
η(CL/*F*)	38.7 (10.7)	37.8 (30.6 to 48.9)	22.6
η(*V_c_*/*F*)	83.5 (12.5)	82.7 (64.1 to 105.0)	42.2
Residual variability (%)			
ε_prop_	32.2 (6.7)	32.1 (28.7 to 35.9)	17.4

a *K_a_*, absorption rate constant; CL, clearance; *V_c_*, central volume of distribution; ε_prop_, proportional residual.

b RSE, relative standard error.

c The successful convergence rate of the bootstrap method for 1,000 resamples was 98.7%.

### Model evaluation.

**FIG 2:**
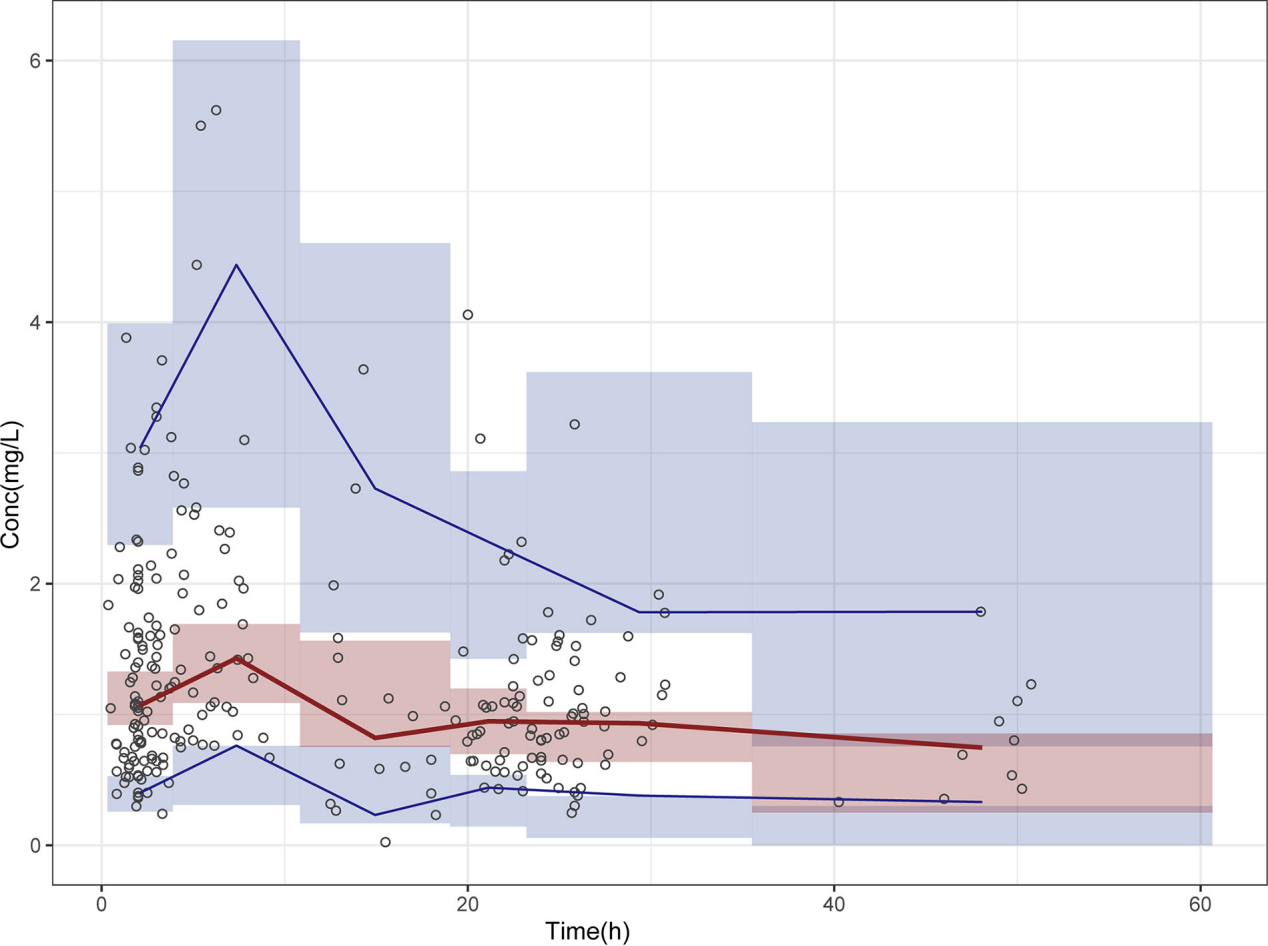
The prediction-corrected visual predictive check. The points represent the corrected observed concentrations, the solid lines represent the 5th, 50th, and 95th percentiles of the corrected observed data, and the blue and red areas represent the confidence intervals for each corrected prediction percentile (at a level of 90%).

The goodness-of-fit plots of the final model showed that observations (OBS) were well correlated with the population prediction (PRED) and individual prediction (IPRED). The conditional weighted residual (CWRES) values were mostly distributed between ±6 and were evenly distributed on the upper and lower sides of the coordinate axis, without apparent bias. The successful convergence rate of the bootstrap method for 1,000 resamples was 98.7%. The estimated PK parameter values from the original data set agreed with the median parameter estimate values from the bootstrap analysis (Table 3). The bootstrap analysis confirmed the robustness of the final model, indicating that the parameter estimation was stable and reliable. A prediction-corrected visual predictive check (PC-VPC) showed that most of the corrected observed concentrations fell within the 90% confidence intervals of the corrected predictive values, and the 90% confidence interval of the typical value predicted by the model basically overlapped the median of the observed value, indicating good predictive performance of the final model (Fig. 2).

### Dosage regimen simulation.

**FIG 3:**
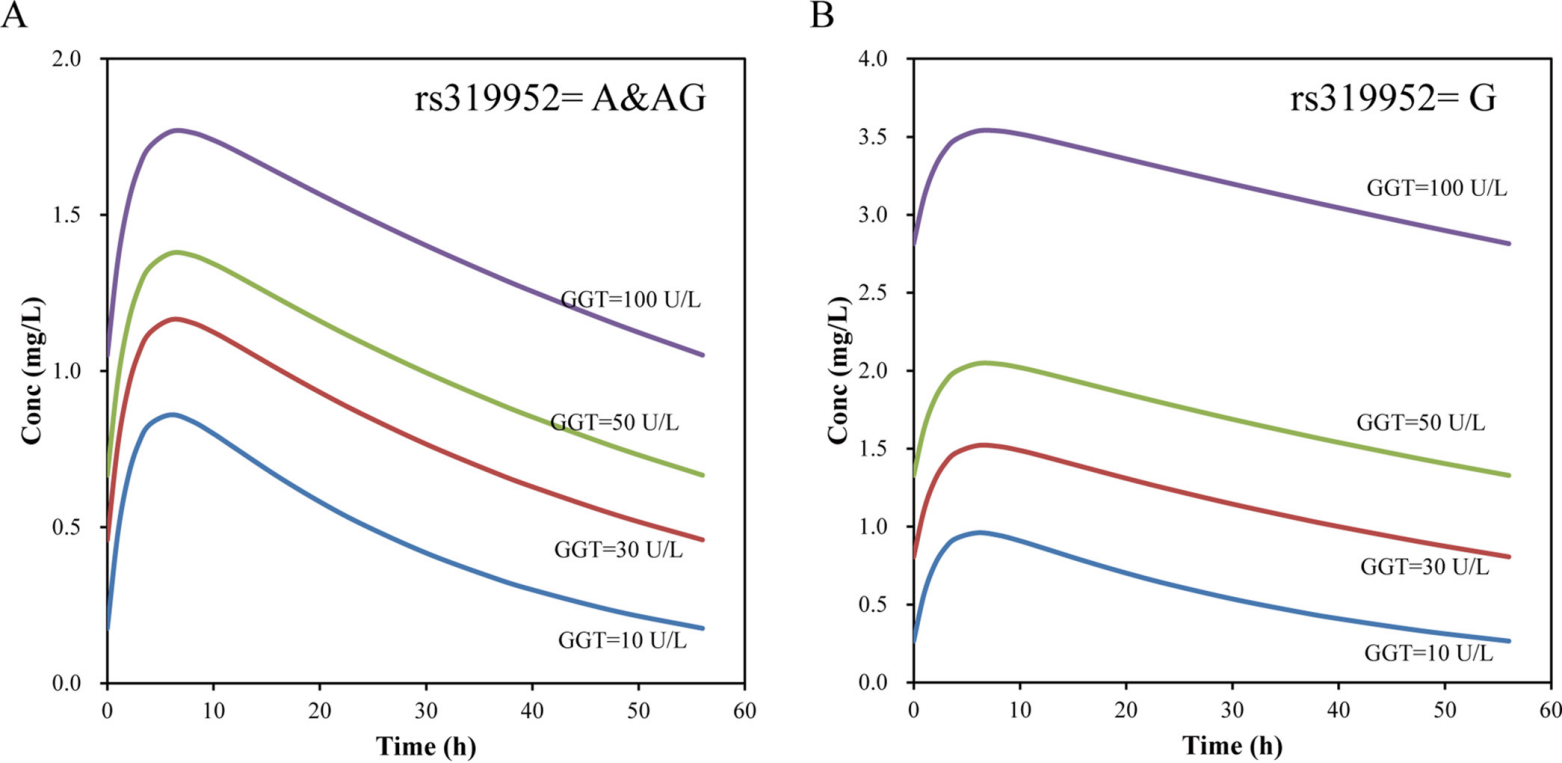
BDQ concentration-time curve with various levels of GGT and different rs319952 genotypes at steady state. (A) rs319952 genotype A&AG; (B) rs319952 genotype G.

Based on the final model, simulations were performed for the typical concentration-time curve with four concentrations of GGT (10 U/L, 30 U/L, 50 U/L, and 100 U/L) and the polymorphism at SNP rs319952 achieving the steady state under an oral dosage of 200 mg three times per week. As shown by the results in Fig. 3A and B, the exposure of BDQ with genotype GG in SNP rs319952 was significantly higher than that with genotypes AA and AG. When GGT achieved 30 U/L, the serum trough concentration (*C*_min,ss_), *C*_max,ss_, and area under the weekly concentration-time curve (AUC_weekly_) of patients with genotype GG were 1.75-fold, 1.3-fold, and 1.46-fold higher, respectively, than those of patients with genotypes AA and AG (Table 4).

**TABLE 4:** BDQ dosage simulation targeting different levels of GGT and rs319952 genotypes

Genotype	GGT concn (U/L)	AUC_weekly,ss_ (mg/L·h)	*C*_max,ss_ (mg/L)	*C*_min,ss_ (mg/L)
A&AG	10	79.746	0.859	0.176
	30	134.529	1.165	0.460
	50	171.561	1.377	0.666
	100	238.620	1.767	1.051
G	10	97.977	0.959	0.266
	30	196.080	1.519	0.806
	50	286.080	2.046	1.328
	100	538.383	3.538	2.815

A PK/PD analysis based on phase IIb data estimates that the half-maximal effective concentration (EC_50_) of BDQ is 1.42 mg/L (95% confidence interval [CI], 1.00 to 2.05) in the continuation phase of bedaquiline treatment (20). With the standard regimen, the trough and maximal concentrations (*C*_min_ and *C*_max_) of BDQ are 0.26 to 0.91 mg/L and 0.9 to 2.1 mg/L, respectively, at the end of the treatment period (200 mg three times per week) (18). In this study, the serum exposure of BDQ increased significantly as the GGT level rose. For the patients with genotype GG in SNP rs319952, those with GGT ranging from 10 U/L to 50 U/L could have *C*_max,ss_ values of 0.959 to 2.046 mg/L, which were within the targeted concentrations, while *C*_max,ss_ increased to 3.538 mg/L in a patient with a concentration of 100 U/L GGT, which was 1.68-fold higher than the highest concentration pursued (Table 4). However, for the patients with genotypes AA and AG, the *C*_max,ss_ values of those with GGT ranging from 30 U/L to 100 U/L fell within the targeted concentrations, except that the *C*_max,ss_ values of patients with 10 U/L GGT were slightly lower than the lowest targeted concentration. Therefore, the dosage simulation indicated that the BDQ concentrations of patients with genotype GG and 100 U/L GGT should be monitored to avoid drug side effects caused by a high plasma concentration of BDQ.

## DISCUSSION

In this work, we successfully developed a population PK model of the first drug in a new class of anti-TB drug, BDQ, among Chinese MDR-TB patients receiving standard BDQ treatment. A one-compartment with first-order-elimination model described the pharmacokinetic parameters of BDQ well. The relationship between GGT and CL/*F* was found to be statistically significant. In addition, this is the first model to include genotype polymorphism in the analysis, which takes advantage of the ability to characterize the covariate relationship between SNPs and BDQ PK, and simulation-based diagnostics indicate that the model robustly captures the observed data.

A four-compartment disposition model of BDQ was developed based on 10 clinical studies, including healthy adults and patients with drug-sensitive TB and MDR-TB, in which the majority of subjects were Black (31.0%) and Caucasian (27.9%) (17). Race and sex were observed to have a significant relationship with apparent clearance (CL/*F*) and apparent central volume of distribution (*V_c_*/*F*). The other study analyzed data from two clinical trials (C208 and C209) and developed a population PK model for BDQ and its metabolite M2 where age, race, body weight, and albumin were significant covariates (18). The estimate of apparent CL (2.62 L/h) was compared to the value presented by the first model (2.78 L/h). Here, we describe the BDQ disposition among Chinese patients by a one-compartment oral absorption model and present a higher value of CL (4.54 L/h), which may be due to the differences in patient race and physical condition. A recent study also based on Chinese MDR-TB patients, in which they collected at least two blood samples at weeks 1 and 2 with 400-mg-daily treatment and detected the plasma concentrations of BDQ, developed a three-compartment model with a transit absorption model for BDQ, but they calculated the smallest value for CL (1.50 L/h) (19). Consistent with the recent model of Chinese patients, body weight was not identified as a significant effect on PK in our work. Additionally, the second model predicts that the highest exposure of BDQ occurs in the first 2 weeks with 400 mg daily, followed by the actual steady state with a lower concentration and a slow accumulation during dosing of 200 mg thrice per week (18). Therefore, the differences in sampling times of the dosing state may be the reason for the large variation of CL/*F* values in these two Chinese studies.

Two covariates were found to be significant in apparent CL. Among the available 87 cases, the frequencies of genotypes AA, AG, and GG in SNP rs319952 (A > G) in the ATP/GTP-binding protein-like 4 gene (*AGBL4*) were 31.31%, 39.39%, and 13.13%, respectively, comparable to the previously published data for the Chinese Han population (43.8%, 44.7%, and 11.5%) (21). *AGBL4*, also known as *CCP6*, encodes the enzyme that participates in controlling assembly, trafficking, and signaling in the microtubules by posttranslational modification of tubulin (22). Our final model showed that the G allele had a 1.4-L/h decrease in CL/*F* compared to that of the AA or AG allele, which indicates that the subjects with the G allele had elevated exposure to BDQ. To our knowledge, there are no other genetic polymorphisms associated with BDQ exposure. However, previous studies proved that *AGBL4* deficiency in mice resulted in enlarged spleens and increased platelet counts with underdeveloped mature megakaryocytes and dysfunctional platelets (23) and also substantially promoted induced pluripotent cell induction and pluripotency of embryonic stem cells (24). In genome-wide association studies (GWAS) in Ethiopian patients, the genetic polymorphisms in *AGBL4*, including the SNP rs319952, were found to be related to antituberculosis drug-induced liver toxicity, although this relationship was not reproduced in the Chinese Han population (21, 25). Therefore, the effect of increased susceptibility to liver toxicity caused by the *AGBL4* genotype on BDQ metabolism needs to be further investigated. Given that BDQ is mainly metabolized by the enzyme CYP3A4 in hepatocytes, it is important to evaluate the effect of hepatic function on BDQ clearance. GGT has been widely used as an important indicator of liver dysfunction and an independent predictor of risk (26). In our model, we first identified that GGT was significantly associated with apparent CL, with a decrease of 30% in CL/*F* per doubling of GGT, suggesting the clinical potential of evaluating BDQ plasma concentration by GGT level.

Dosage simulation based on the final model demonstrated that the disposition of BDQ of patients with allele GG in SNP rs319952 was considerably higher than that with alleles AA and AG. As a GGT concentration of 30 U/L was achieved, patients with allele GG had *C*_min,ss_, *C*_max,ss_, and AUC_weekly,ss_ values 1.75, 1.3, and 1.46 times higher than those of patients with alleles AA and AG (Table 4). Additionally, with increasing GGT concentrations, the plasma exposures of BDQ were markedly elevated. Taking patients with alleles AA and AG as examples, *C*_min,ss_, *C*_max,ss_, and AUC_weekly,ss_ calculated from the 100 U/L GGT population were increased up to 5.97, 2.06, and 2.99 times, respectively, compared to those from the 10 U/L GGT population (Table 4). As the *C*_max,ss_ of allele GG with 100 U/L GGT exceeded the highest target pursued (2.1 mg/L) by 1.68 times, the plasma exposure of BDQ under this condition needs special attention and monitoring.

In conclusion, we combined the related genotype polymorphisms to establish a population PK model for BDQ among Chinese MDR-TB patients. To our knowledge, this is the first study to identify the genetic polymorphisms in SNP rs319952 in the *AGBL4* gene and glutamyl transferase as having a significant impact on BDQ clearance. Based on the final model, we simulated the BDQ exposure according to different alleles of rs319952 and GGT concentrations. The impact of covariates on BDQ PK might provide potential clinical predictions for BDQ concentration by the rs319952 allele and GGT level in individualized medication.

## MATERIALS AND METHODS

### Ethics.

Written informed consent was signed by all patients, and the study was approved by the Medical Research Ethics Committee of Shenzhen Third People’s Hospital (reference no. 2021-003).

### Study subjects.

BDQ plasma concentration data were obtained from a prospective, open-label, population pharmacokinetic study conducted on MDR-TB patients in the Third People’s Hospital of Shenzhen, China. The demographic characteristics for each patient were collected, and the laboratory examination results 2 days before or after PK sampling were used for analysis.

### Dosage and blood sampling.

All subjects received the tablet formulation with the approved regimen, 400 mg daily for the initial 2 weeks of treatment, followed by 200 mg three times per week for another 22 weeks. The other background drugs used included cycloserine (*n* = 91, 91.92%), linezolid (*n* = 96, 96.97%), pyrazinamide (*n* = 49, 49.49%), moxifloxacin (*n* = 41, 41.41%), clofazimine (*n* = 34, 34.34%), levofloxacin (*n* = 27, 27.27%), and ethambutol (*n* = 1, 1.01%). Blood samples were taken during weeks 3 to 24 (when patients were receiving the 200 mg three times per week dosage) and collected in EDTA-containing tubes. Samples were centrifuged within an hour at 3,000 × *g* at 4°C for 10 min, and then plasma was stored at −80°C.

### BDQ assay.

Plasma samples were used for BDQ quantification by liquid chromatography-tandem mass spectrometry (LC-MS/MS) after protein precipitation. Fifty microliters of plasma was deproteinized with 150 μL of precooled methanol containing internal standards. After centrifugation, the supernatant was analyzed by LC-MS/MS (LCMS-8040 CL; Shimadzu, Japan) with an electrospray ionization (ESI) source in positive ion mode. An ACE Excel C_18_-PFP column (2-μm particle size, 2.1 × 50 mm) was used for bedaquiline separation with a flow rate of 0.5 mL/min and column temperature of 45°C. The mobile phases consisted of water containing 0.1% formic acid (vol/vol) (mobile phase A) and acetonitrile/methanol (1:1) containing 0.1% formic acid (vol/vol) (mobile phase B). The gradient elution of 1% B was kept for 0.5 min and then changed linearly to 85% B to 98% B to 1% B during 3.1 min and maintained for 2.0 min. Multiple reaction monitoring (MRM) was used to monitor bedaquiline and the internal standards, with bedaquiline at *m/z* 555.2 to 58.0, internal standard at *m/z* 559.2 to 62.0, and collision energy (CE) at −25 V for bedaquiline and the internal standard. The mass parameters were as follows: nebulizer gas flow, 3.0 L/min, drying gas flow, 18 L/min, desolvation line (DL) temperature, 250°C, heating block temperature, 400°C, and interface voltage, 4.5 kV. Bedaquiline and the internal standard were purchased from Toronto Research Chemical (TRC). The range of the assay was 0.024 to 7.61 μg/mL.

### SNP selection and genotyping.

Genomic DNA was extracted from the peripheral blood samples using a nucleic acid isolation kit (magnetic beads) (DaAn Gene Co., Guangzhou, China). SNP genotyping was performed on the MassArray platform (Agena Bioscience) combined with the iPlex assay (Sequenom, Inc., Hamburg, Germany) and matrix-assisted laser desorption ionization-time of flight (MALDI-TOF) mass spectrometry. Ten nanograms of genomic DNA of each sample was standardized for genotyping. The DNA samples were amplified by multiplex PCR and treated with shrimp alkaline phosphatase, and then the products were subjected to a specific multiplex single-base extension PCR. The extension products were desalted using clean resin (MassArray clean resin, 28 g) and then transferred onto a SpectroChip array (Sequenom, Inc., Hamburg, Germany), where they were crystalized with a prespotted MALDI matrix. The alleles were distinguished by MALDI-TOF mass spectrometry. Data were processed and analyzed automatically using Typer software (MassArray Typer 4.1.0.83).

### Population pharmacokinetic modeling of BDQ.

The BDQ concentration data were fitted by the one- or two-compartment with first-order-elimination model. An exponential model (equation 3) was used to account for interindividual variability (IIV), as follows:

(3) $$P_{i}=P_{\text{typical}}\times \exp(\eta_{i})$$

where *P_i_* represents the individual PK parameter, *P*_typical_ represents the typical population value of the PK parameter, and η*_i_* represents the individual variability, which is normally distributed with a mean of zero and variance of ω^2^.

Residual unexplained variability was tested by an additive model (equation 4), a proportional model (equation 5), or a combined additive and proportional model (equation 6), as follows:

(4) $$C_{\text{obs},\mathrm{ij}}=C_{\text{pred},\mathrm{ij}}\;+\;\varepsilon_{\mathrm{ij},\text{add}}$$

(5) $$C_{\text{obs},\mathrm{ij}}=C_{\text{pred},\mathrm{ij}}\times (1+\varepsilon_{\mathrm{ij},\text{prop}})$$

(6) $$C_{\text{obs},\mathrm{ij}}=C_{\text{pred},\mathrm{ij}}\times (1+\varepsilon_{\mathrm{ij},\text{prop}})\;+\;\varepsilon_{\mathrm{ij},\text{add}}$$

where *C*_obs,_*_ij_* and *C*_pred,_*_ij_* represent the observed values and the predicted values for plasma concentration, respectively, and ε*_ij_*_,add_ and ε*_ij_*_,prop_ represent the additive and proportional residuals, which were normally distributed with a mean of zero and variance of σ_1_^2^ and σ_2_^2^, respectively.

A covariate model was developed to evaluate the impact of covariates on the parameter. Continuous covariate relationships were modeled by equation 7, and categorical covariates were modeled using equation 8, as follows:

(7) Ptot=Ptypical×(COV/COVmedian)^θ

(8) $$P_{\text{tot}}=\{\begin{array}{c} P_{\text{typical}}\\ P_{\text{typical}}\times \left(1\;+\text{θ}\right)\; \end{array}\;\begin{array}{c} \text{if COV}=0\\ \text{if COV}=1 \end{array}$$

where *P*_typical_ represents the typical population value of the PK parameter, COV_median_ represents the median value of the covariate, and θ represents a scale factor for the covariate.

We first used the graphical method to analyze the correlation between the covariates and PK parameters (CL/*F* and *V_c_*/*F*) and initially screened out the significantly correlated covariate/parameter relationships (*P* < 0.05). If a subject’s continuous covariate was missing, it was filled with the median of the covariate, and if a subject’s categorical covariate was missing, it was filled with the classification with the highest frequency of occurrence. Then, the significantly correlated covariate/parameter relationships were further screened into the final model using a forward inclusion and backward elimination method. The forward inclusion and backward elimination steps were conducted at significance levels of α = 0.01 and α = 0.005, respectively, using the likelihood-ratio test. We also considered the pharmacological plausibility in the selection of the covariates.

The minimum value of objective function values (OFVs) served as a guide during model building. Model selection was also based on parameter plausibility and diagnosis plots. The goodness-of-fit for the final model was evaluated using diagnostic graphs. A nonparametric bootstrap analysis with 1,000 times resampling and replacement was performed, and the parameters that converged successfully were counted to obtain their medians and 95% confidence intervals. The prediction performance was evaluated through the PC-VPC (27). Based on the final population PK model, we simulated the typical BDQ exposure in steady state with different covariates under the standard dose regimen and calculated the PK parameters, such as *C*_min,ss_, *C*_max,ss_, and AUC_weekly,ss_, to estimate the impact of covariates on BDQ exposure. The steady-state concentration was simulated by setting the SS Item in the NONMEM data file.

### Software.

Model development and simulation were performed using NONMEN 7.4 (level 1.0, ICON Development Solutions, USA). The first-order conditional-estimation method with interaction was used to estimate model parameters. A bootstrap procedure was performed using Wings for NONMEN (WFN741; http://wfn.sourceforge.net). R software (version 3.6.3) was used to process the output data, statistical analysis, and plotting.
